# A Resorbable Antibiotic-Eluting Polymer Composite Bone Void Filler for Perioperative Infection Prevention in a Rabbit Radial Defect Model

**DOI:** 10.1371/journal.pone.0118696

**Published:** 2015-03-27

**Authors:** Benjamin D. Brooks, Kristofer D. Sinclair, David W. Grainger, Amanda E. Brooks

**Affiliations:** 1 Department of Pharmaceutics and Pharmaceutical Chemistry, University of Utah, Salt Lake City, Utah, 84112–5820, United States of America; 2 Department of Bioengineering, University of Utah, Salt Lake City, Utah, 84112–5820, United States of America; 3 Elute Inc., 417 Wakara Way, Suite 3510, Salt Lake City, Utah, 84108, United States of America; Institute for Frontier Medical Sciences, Kyoto University, JAPAN

## Abstract

Nearly 1.3 million total joint replacement procedures are performed in the United States annually, with numbers projected to rise exponentially in the coming decades. Although finite infection rates for these procedures remain consistently low, device-related infections represent a significant cause of implant failure, requiring secondary or revision procedures. Revision procedures manifest several-fold higher infection recurrence rates. Importantly, many revision surgeries, infected or not, require bone void fillers to support the host bone and provide a sufficient tissue bed for new hardware placement. Antibiotic-eluting bone void fillers (ABVF), providing both osteoconductive and antimicrobial properties, represent one approach for reducing rates of orthopedic device-related infections. Using a solvent-free, molten-cast process, a polymer-controlled antibiotic-eluting calcium carbonate hydroxyapatite (HAP) ceramic composite BVF (ABVF) was fabricated, characterized, and evaluated *in vivo* using a bacterial challenge in a rabbit radial defect window model. ABVF loaded with tobramycin eliminated the infectious burden in rabbits challenged with a clinically relevant strain of *Staphylococcus aureus* (inoculum as high as 10^7^ CFU). Histological, microbiological, and radiographic methods were used to detail the effects of ABVF on microbial challenge to host bone after 8 weeks *in vivo*. In contrast to the HAP/BVF controls, which provided no antibiotic protection and required euthanasia 3 weeks post-operatively, tobramycin-releasing ABVF animals showed no signs of infection (clinical, microbiological, or radiographic) when euthanized at the 8-week study endpoint. ABVF sites did exhibit fibrous encapsulation around the implant at 8 weeks. Local antibiotic release from ABVF to orthopedic sites requiring bone void fillers eliminated the periprosthetic bacterial challenge in this 8-week *in vivo* study, confirming previous *in vitro* results.

## Introduction

Global markets for orthopedic procedures and products are estimated to reach $41.8 billion by 2016 based on an anticipated annual growth rate of 7.4% from 2009–2016 [1,2]. This growth can be attributed to increased applications in aging global populations and in new patient groups from developing countries [1,3]. Major contributors to this growth are revision Total Joint Replacements (TJRs), including Total Hip Arthroplasty (THA) revisions projected to exceed 50,000 annually by 2030 and Total Knee Arthroplasty (TKA) revisions projected to number nearly 250,000 procedures by 2030 [4]. Despite the success of TJRs in improving quality of life, up to 10% [5] of these implanted devices fail, requiring revision procedures [6] at a cost of $42,000-$56,000 per revision procedure (U.S.) [7]. Despite consistently low failure rates (~1–3%) for primary TJR procedures, TJR infection persists as a significant cause of implant failure, recurrent failure, and skyrocketing treatment costs. Infection recurrence rates in revision surgeries are anticipated to increase drastically to 20–30% [8,9]. Rising numbers of TJRs combined with finite infection rates have yielded dramatic growth in numbers of patients worldwide suffering with these devastating infections [10–12]. Consequently, infection prevention and treatment both continue to be ongoing foci of the orthopedic community.

To address this increasing healthcare threat, current therapeutic interventions for periprosthetic infections include: 1) systemic intravenous or oral antibiotic therapy, 2) surgical intervention (e.g., irrigation and debridement, resection arthroplasty, and/or revision arthroplasty), and 3) local antibiotic therapy (e.g., antibiotic loaded bone cement (ALBC) and ALBC spacers as both permanent and temporary implants). Unfortunately, each approach suffers limitations and clinical management of these complex infections often includes a mixture of therapies, techniques, and antibiotics. Systemic long-term antibiotic treatment often fails due to poor tissue site biodistribution as well as combinations of systemic side effects, poor patient compliance, and the development of drug-resistant pathogens [13–15]. Surgical interventions place additional financial, temporal, occupational, or other life-style burdens on the patient and do not guarantee infection elimination. Finally, local antibiotic therapy (e.g., bone cement spacers and beads) has demonstrated the ability to reduce periprosthetic infection rates; however, this approach also possesses limitations. ALBC (e.g., Palacos G from Biomet, DePuy 1 from DePuy Orthopaedics, Cemex Genta from Exatech, Versabond AB from Smith & Nephew, or Simplex P from Stryker Orthopaedics), the most common local antibiotic-delivering device for orthopedic applications, is regulated in the U.S. by the FDA for use in total joint arthroplasty revision surgeries, lacking adequate drug release kinetics to protect the entire device [16–22]. Moreover, after an initial drug-releasing burst phase, antibiotic concentrations quickly decline and antibiotic leaches at sub-therapeutic levels for months to years [10,19]. This not only facilitates antibiotic resistance at the implant site, but also allows any unretrieved bone cement to act as a foreign body and nidus for recurrent infection [23,24]. Therefore, new antimicrobial strategies that effectively reduce known complications associated with revision TJR procedures are a compelling need.

While surgical TJR procedures, including THA and TKA, account for the majority of bone void filler (BVF) utilization, a large percentage of the more than two million orthopedic procedures performed annually in the U.S. use BVFs for repair of traumatic injury or other boney defects [25–28]. Significantly, a substantial fraction of secondary non-cemented revision TJR procedures require bone graft to support host tissue implant integration. Consequently, the demand for BVFs is estimated at ~$1.3 billion within an expanding $29 billion total orthopedics biomaterial market [25,29,30]. Estimates put the BVF market for knee reconstruction alone at $600 million by 2030 with an additional $150 million for THA (estimated from an average BVF volume per revision of 12.7 ml and ~$1,800 per surgery); the future need for BVF with reliable osseointegration and antimicrobial properties is therefore substantial [3,31]. A successful BVF will be biologically active, possessing the following characteristics: biocompatibility (tissue-benign with minimal foreign body response), bioresorbability, and osseointegrating (integration with host bone) [5,13,26]. Given the high incidence of infections in TJR revisions and the prevalence of bone graft in these procedures, a resorbable antibiotic-eluting BVF is considered an attractive clinical option [5,28].

The hypothesis driving this work is that an implanted resorbable antibiotic-eluting BVF with extended drug release capability reliably prevents bone infection while facilitating bone integration and eventual resorption. Previous reports [13, 32] utilizing this polymer-controlled, antibiotic-releasing BVF demonstrated the ability to eliminate high bacterial inocula (e.g., 10^7^ colony forming units (CFU) per milliliter) in *in vitro* assays [13] beyond 8 weeks. Thus, based on extensive *in vitro* characterization, a BVF composite comprising synthetic highly porous inorganic granules combined with other clinically familiar degradable components (e.g., both inorganic and polymer phases) [33–37] should provide appropriate pharmacokinetics to eliminate a periprosthetic bacterial challenge and provide a resorbable, osteoconductive scaffold to facilitate host tissue integration in a rabbit radial window defect model. The hypothesis was investigated using the following aims: 1) assess *in vivo* antimicrobial efficacy and 2) determine the suitability of the antibiotic-eluting BVF to provide an osteoconductive matrix to support active bone remodeling using an established rabbit radial window defect model [23, 27]. These aims were evaluated using quantitative and qualitative assessments of histology, radiography, and microbiology. Here we report the first phase of an extensive *in vivo* study designed to follow on previous *in vitro* testing [13,25,32]

## Methods

### Antibiotic-Eluting BVF Fabrication

Antibiotic-eluting BVF devices were fabricated as previously described [32]. Briefly, granular ceramic commercial bone grafting biomaterial (ProOsteon 500R —a hydroxyapatite (HAP) calcium carbonate-based hybrid coralline bone void filler, Biomet, USA—HAP/BVF hybrid) was morselized and sieved to 150–425 μm. Polycaprolactone (~24% w/w, 10 kDa, PCL, Sigma 440752) and polyethylene glycol (~3% w/w, 20 kDa, Sigma P2263) were mixed and melted at 75°C. Subsequently, morselized ProOsteon 500R hydroxyapatite coralline (HAP) granules (~63% w/w) and tobramycin sulfate powder (~10% w/w, Research Products International) were mixed into the molten two-component polymer slurry. Table 1 summarizes this implant compositional design for each implant cohort. The molten bone void filler mixture was compressed into a single silicone mold containing twelve 2 mm x 2 mm x 6 mm rectangular cutouts. The resulting rectangular BVF implants were allowed to solidify at room temperature and then quality control-assessed as described previously [32]. All BVF implants were sterilized via Sterrad sterilization, a low temperature hydrogen peroxide gas plasma processing to preserve both polymer integrity and antibiotic activity prior to implantation.

**Table 1:** Cohort designs for rabbit radial window defects implanted with bone graft with or without tobramycin and with or without *S*. *aureus*.

**Cohort**	**Implant Description**	**Infectious Dose**	**Morbidity/Infection**	**Time to Sacrifice (weeks)**	**Cohort Purpose**
1	HAP/BVF hybrid	None	No infection	8	Surgical control
2	HAP/BVF hybrid	10^5^ CFU	Massive local infection	2–4	Infection model validation
3*	HAP/BVF + PCL/PEG polymers, no antibiotic	None	No infection	8	Safety of the polymers
4	HAP/BVF + PCL/PEG polymers, no antibiotic	10^5^ CFU	Signs of a local infection, self healed	8	Safety and antimicrobial efficacy of polymers
5	HAP/BVF hybrid, 10% antibiotic soak	10^5^ CFU	Signs of a local infection, self healed	8	Current clinical practice control
6*	HAP/BVF + PCL/PEG polymers + tobramycin antibiotic	None	No infection	24	Osseointegration Control
7	HAP/BVF + PCL/PEG polymers + tobramycin antibiotic	10^7^ CFU	No observable infection	8	Test device

* Data not presented in this manuscript.

### Cohort Experimental Design

Each cohort included seven animals according to a power analysis and as supported by literature [34,38]. For Cohorts 1, 2 and 5, the ProOsteon 500R HAP/BVF hybrid was not granulated and instead was shaved to be the same dimensions (~2 mm x 2 mm x 6 mm) as the antibiotic-eluting BVF polymer composite devices. Cohort 1 did not include any antibiotic, polymer, or infectious inoculum to assess the sterility of the surgical procedure. Analogous with the Cohort 1 implant, Cohort 2 did not include any antibiotic or polymer but did include an infectious inoculum to assess the ability of the experimental model to produce a propagating bone infection in the presence of the BVF/HAP device substrate. Cohort 2 was designed to represent the current clinical standard for filling bone voids. To serve as a direct challenge to Cohort 2 the antibiotic- eluting bone void filler (Cohort 7) was developed to reduce the rate of device related infection, associated with these procedures, as well as act as a bone void filling material. No antibiotic was utilized in Cohorts 3 and 4 to assess the safety of the polymer components of the device both in the absence (Cohort 3) and the presence (Cohort 4) of an infectious inoculum. Cohort 5 implants, representative of a current clinical practice comparator, were not a granulated HAP/BVF hybrid but instead a larger “shaved” fragment of HAP/BVF soaked in a 10% tobramycin saline solution for 10 minutes immediately prior to implantation. Cohort 6 was implanted with the polymer-controlled, antibiotic-eluting BVF/HAP composite device but did not include an infectious inoculum. This cohort was also extended to 24-weeks to allow sufficient time for cellular infiltration and host bone ingrowth (i.e. osseointegration). Cohort 7 (test cohort) was designed to assess the antimicrobial efficacy of the polymer-controlled, antibiotic-eluting BVF/HAP composite device by including a rigorous infectious challenge. This test cohort was subsequently compared to Cohort 2 (isolated the BVF/HAP hybrid component of the device), Cohort 4 (isolated the BVF/HAP hybrid substrate with the polymer components), and Cohort 5 (representative of current clinical practice—release of drug from BVF/HAP hybrid in the absence of the polymer barrier).

### Bacterial Strain

All bacterial inocula and *in vitro* microbial methods used *Staphylococcus aureus* (*S*. *aureus*) strain UAMS-1 (ATCC strain 49230) as previously described [13,34,32,39]. For preparation of bacterial inoculum, the strain was grown overnight on 5% sheep blood agar at 37°C. Isolated colonies were suspended in sterile saline to an optical density of 10^5^ (Cohorts 2, 4, and 5) or 10^7^ (Cohort 7) CFU in 50 μL using a nephelometer (BD Biosciences, USA). Note that Cohort 7 (test device) was inoculated with 100-fold larger CFU to provide a more rigorous infectious challenge.

### *In Vivo* Rabbit Radial Infection Model

**Fig 1:**
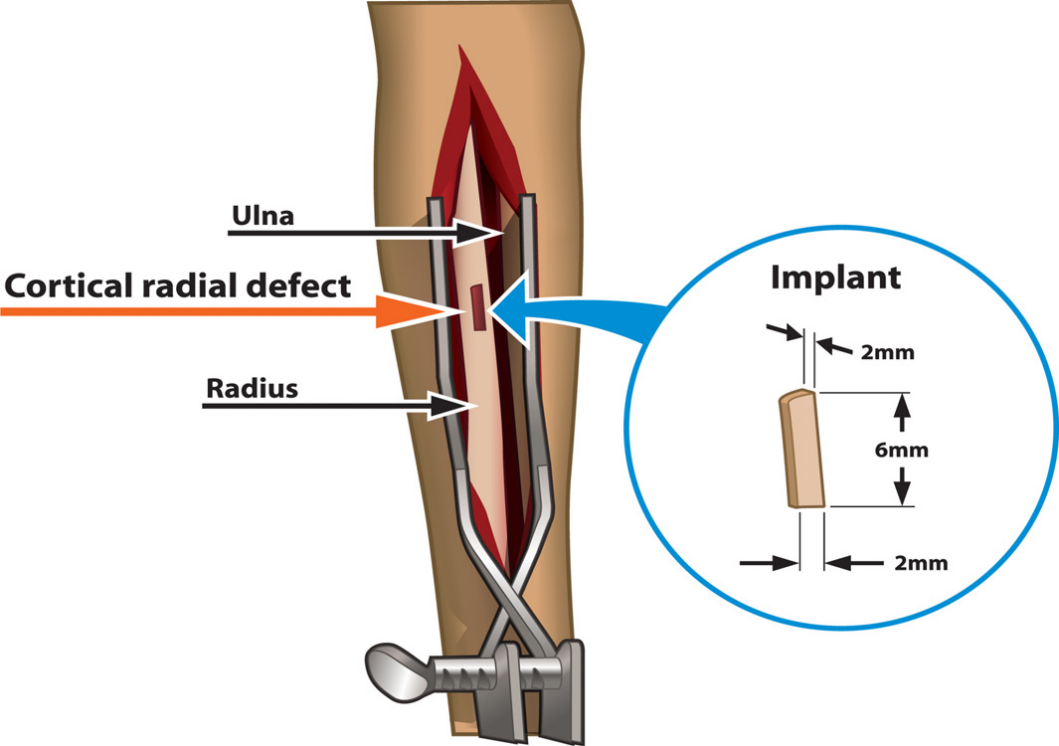
Schematic describing the surgical procedure for the rabbit radial BVF implant.

All experiments were carried out in accordance with the guidelines of the Institutional Animal Care and Use Committee at the University of Utah (Center for Comparative Medicine, an AAALAC-approved facility, University of Utah) under the direction of skilled veterinary staff. Seven cohorts (n = 7) (Table 1) of skeletally mature New Zealand white rabbits (3–4 kg) were used to isolate each variable of the new drug delivery system to assess the antimicrobial efficacy of the device (see Cohort Experimental Design and Table 1). Rabbits were individually housed in temperature-controlled cages at 30–40% humidity under a 12-hour light/dark cycle and provided food and water *ad libitum*. The surgical procedure was adapted from prior protocols of Koort et al. [38] and Smeltzer et al. [39]. Briefly, each rabbit was weighed, body temperature was recorded, and the rabbits were anesthetized with isofluorane for the duration of the surgery. The medial surface of the right forelimb was clipped and scrubbed with a povidone iodine surgical scrub solution (Medline, USA) followed by three wipes with 70% ethanol and a final povidone iodine surgical scrub solution to create a sterile field. Subsequently, the surgical site was draped and the medial surface of the radius was surgically exposed. A transcortical defect (~6 mm by 2 mm by 2 mm) was drilled using a burr with saline irrigation into the proximal medial metaphysis of the right radius (Fig. 1). The local bone marrow was removed by saline lavage and 10^5^ (Cohorts 2, 4, and 5, Table 1) or 10^7^ (Cohort 7, Table 1) CFU of *S*. *aureus* was injected into the medullary canal, proximal to the window defect. No bacteria were used in control cohorts (Cohorts 1, 3 and 6, Table 1). The surgical defect was then filled with the antibiotic-eluting BVF (each device was approximately 42 mg). The sterilized 2 mm x 2 mm x 6 mm antibiotic-eluting BVF device was press-fit into the drilled bone window defect and the soft tissue was closed over the defect with 3.0 silk sutures. When placed, the BVF device appeared to have only minimal penetration into the marrow space and was clearly visible in the surgical site prior to soft tissue closure. A fur patch at the nape of the neck was clipped and a fentanyl patch applied for post-operative pain control. Additionally, buprenorphine was administered subcutaneously (0.03 mg/kg) for immediate analgesic effects. The dosage was repeated at 8–10 hour intervals 2–3 times as indicated for pain management or prescribed by veterinary staff. Perioperative antibiotics, usually the standard of care, were withheld due to study objectives. Rabbits were monitored daily for signs of pain or distress and examined weekly under isofluorane anesthesia throughout post-operative recovery. Weekly examinations included radiographs, temperature and weight recordings, wound site evaluation and scoring, and blood microbiology for systemic infection determination. Rabbits exhibiting symptoms of infection including altered body temperature, suppressed appetite, purulent discharge from the wound site, radiographic evidence of progressive osteomyelitis, and/or altered gait were removed from the study prior to the study endpoint.

### Euthanasia

Rabbits were evaluated using a modification of the Checketts’ grading scale for bone health (Table 2) and a modification of the Petty grading scale for infection (Table 3) as well as recommendations of veterinary staff [40–42]. Animals were euthanized if a composite score of greater than 2.5 was observed, using NIH guidelines for the humane treatment of animals as well as approved in University of Utah IACUC protocol 12–01004. Animals were euthanized in cases of blood-borne infection, overwhelming localized infection, excessive signs of distress, appetite suppression, limited water consumption, lethargy, or lameness. Briefly, animals were anesthetized with isofluorane prior to injection of beuthanasia (1 ml / 4.5 kg).

**Table 2:** Modified Checketts’ scale for bone health [42].

**Parameter**	**Score**	**Description**
Intraosseous acute inflammation	0	Normal/Not present
	1	Minimal to mild inflammation with no intramedullary abscess
	2	Moderate to severe inflammation with no intramedullary abscess
	3	Minimal to mild inflammation with intramedullary abscess
	4	Moderate to severe inflammation with intramedullary abscess
Intraosseous chronic inflammation	0	Normal/Not present
	1	Minimal to mild chronic inflammation with no significant intramedullary fibrosis
	2	Moderate to severe chronic inflammation with no significant intramedullary fibrosis
	3	Minimal to mild chronic inflammation with significant intramedullary abscess
	4	Moderate to severe chronic inflammation with significant intramedullary abscess
Periosteal inflammation	0	Normal/Not present
	1	Minimal to mild inflammation with no subperiosteal abscess formation
	2	Moderate to severe inflammation with no subperiosteal abscess formation
	3	Minimal to mild inflammation with subperiosteal abscess formation
	4	Moderate to severe inflammation with subperiosteal abscess formation
Bone necrosis	0	Normal/Not present
	1	Single focus of necrosis without sequestra formation
	2	Multiple foci of necrosis without sequestra formation
	3	Single focus of sequestra
	4	Multiple foci of sequestra

**Table 3:** Modified Petty scale to assess infection (i.e., external inflammation, soft tissue inflammation, bone necrosis, and osteomyelitis) during post-operative monitoring [42].

**Score**	**Modified Petty Score**
0	No evidence of infection
1	Infection involving the skin and subcutaneous skin only (Mild) Local swelling, local tenderness, local warmth, erythema >0.5-2cm around site
2	Infection with erythema >2cm plus one of the following (moderate), swelling, tenderness, warmth or discharge
3	Infection with erythema >2cm plus infection of the deeper tissues (severe)
4	Any local infection with evidence of two metabolic perturbations (Critical)

### Radiography

Pre- and post-operative radiographs were captured as well as weekly images from both anteroposterior and lateral views using an OEC Miniview 6600 (GE Healthcare). Infection indicators were assessed on the basis of evidence: (a) inflammation, (b) bone necrosis, (c) widening of the bone shaft, (d) formation of new bone, and (e) deformation of soft tissue [39].

### Post-Operative Microbiology

Following euthanasia and dissection of the surgical site, aseptic swabs of the soft tissue and of the implant site in the bone were taken. Swabs were cultured on 5% sheep blood agar plates for 24 hours to 72 hours at 37°C.

### Bone Histological Processing

Post-euthanasia, each implant site was dissected for fixation. Importantly, soft tissue was dissected from the bone and processed independently. Bone samples were cut no thicker than 3–5 mm to ensure fixative penetration. All steps of the fixation and dehydration process occurred at room temperature at 20 times the volume of the sample [41]. Both bone and soft tissue samples were placed in 10% Neutral Buffered Formalin (NBF) (Fisher, USA) for 3 days. The fixative was changed and replaced twice for another 3 days. The samples were then placed in 70% ethanol (EtOH) for 2–3 days. The samples were rinsed for 10 minutes in deionized (DI) water. The 70% EtOH step was repeated three times for 24 hours each. The samples were then placed in 95% EtOH three times for 24 hours each. The sample was then placed in 100% EtOH three times for 24 hours. After fixation, bone samples were infiltrated by methyl methacrylate (MMA) under vacuum at 4°C. The fixed bone samples were placed into a 200 ml container of 80% methyl methacrylate (MMA, Polysciences, USA) and 20% n-butyl phthalate (plasticizer, Sigma Aldrich, USA) for five days. Next, bone samples were infiltrated sequentially by placing the sample into a 200 ml container of: 1) 80% MMA and 20% n-butyl phthalate and 0.1 grams of benzoin methyl ether (Polysciences, USA) for seven days and 2) 80% MMA and 20% n-Butyl phthalate and 0.2 grams of benzoin methyl ether for 9 days. After infiltration, bone samples were placed in an ultraviolet (UV) light-transparent container and embedded to a 2–3 cm depth of a solution of 80% MMA and 20% n-butyl phthalate and 0.2 grams of benzoin methyl ether and allowed to polymerize under UV exposure. The embedding step was repeated until the sample was completely encased in hard poly(methyl methacrylate) (PMMA) with each PMMA layer being allowed to cure prior to the next layer being added. Samples were cut into 1 mm thick sections using a bone saw (Isomet Low Speed Buehler Saw) and mounted on plastic histology slides after which they were ground and polished to optical transparency (~50–70 μm).

### Histological Staining

Each section was ground (400 grit sand paper) and polished (0.3 μm aluminum silicate polisher) to visual clarity. Three sections from each sample were stained using a Sanderson’s rapid bone stain (Dorn and Hart Microedge, USA) with an acid fuchsin counter stain according to the manufacturer’s protocol. Briefly, Sanderson Rapid Bone Stain (SRBS) and a water wash were heated to 55°C. SRBS was applied to sections for 2 minutes after which the slide was rinsed in water wash (20 seconds). Slides were not dried and the acid fuchsin was applied for 15–30 seconds followed by room temperature water wash for 20 seconds. Slides were visualized using either a Nikon Eclipse TE2000-U with a 12-bit Retiga Exi color camera (QImaging, USA) and QCapture software or the Zeiss Axio scan.Z1 with Zen software to stitch the images together. Images were captured at a 2x-20x magnification.

### Histological Scoring

Histology was scored (scale of 0 to 4) by three independent and blinded investigators under light microscopy for: 1) capsule thickness (4 = <50 μm; 0 = >250 μm), 2) inflammatory response (4 = no inflammatory response; 0 = moderate to severe inflammation with abscess), 3) cellular infiltration (4 = mostly bone; 0 = dense tissue and exclusively inflammatory cells), and 4) qualitative bone reaction (4 = similar appearance to host bone; 0 = dead bone) according to Sinclair’s histomorphological scale [40]. All scores from a single cohort were averaged for each metric.

### Statistical Methods

Based on pilot studies, seven rabbits per cohort were necessary for statistical significance (an expected difference of 2.0 in the ratio for the antimicrobial cohort versus the control cohort with no antibiotics, a standard deviation for both cohorts of 1.0, a type I error of 0.05, and power of 80% [43]). Pairwise one-way ANOVA was used to determine significant differences between cohorts (p ≤ 0.05) based on the semi-quantitative scoring of Sanderson stained histology slides.

## Results

To corroborate previous *in vitro* findings of extended antimicrobial efficacy [13,32], antibiotic-eluting BVF polymer composite implants were manufactured as described previously [32] and implanted into a cortical window defect in an established rabbit radial model (Fig. 1) [38,39]. Antibiotic-eluting polymer-controlled BVF/HAP composite devices were easily fabricated in controlled dimensions, readily handled and compressed into the molds, shaped and carved for facile perioperative manipulation in diverse bone site dimensions and geometries. Each component of the BVF polymer composite was isolated and investigated to assess the specific *in vivo* response to the BVF (Cohorts 1 and 2), the polymer component (Cohorts 3 and 4), the surgical procedure (Cohort 1), normal host bone healing (Cohort 1), and the antibiotic-eluting BVF polymer composite (Cohorts 6 and 7). Animals were divided into seven statistically significant cohorts and evaluated for morbidity and mortality, to assess both safety and antimicrobial efficacy. Cohorts are described in the materials and methods above and in Table 1.

### Morbidity and Mortality Scoring

**Fig 2:**
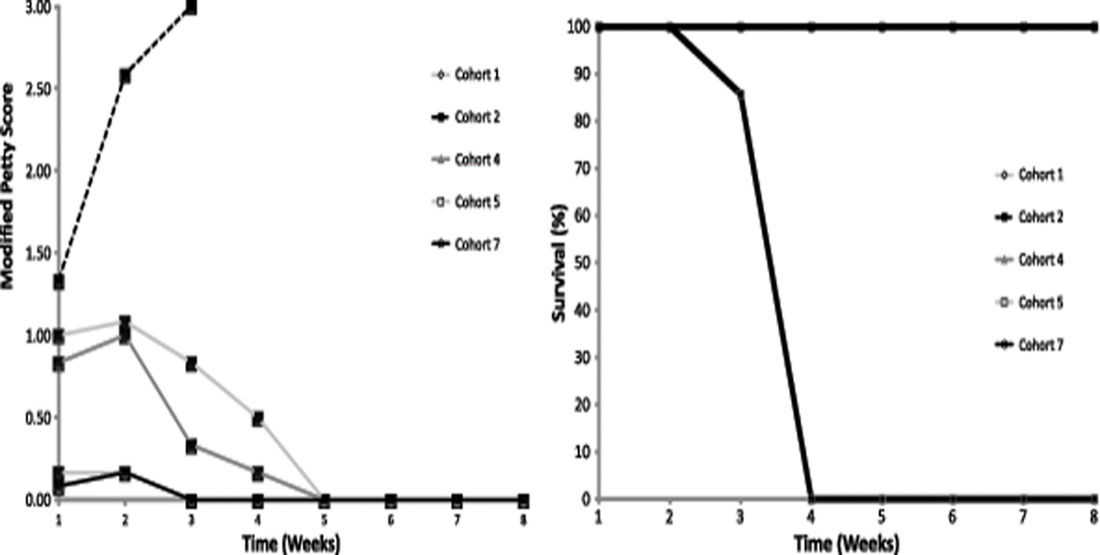
A) Rabbit morbidity scores plot and B) Kaplan-Meier survival plot post-surgery and treatment. Note that in 2B, graphs for Cohorts 1, 4, 5, and 7 are completely overlaid, indicating 100% survival in all of these cohorts. In both 2A and 2B, only infected cohorts are shown with the exception of Cohort 1, which was included as a non-infection comparator.

Morbidity and mortality were monitored for eight weeks (Fig. 2). Morbidity was scored according to the modified Checketts’ and Petty scales shown in Tables 2 and 3 [40]. Importantly, the higher the score the more adverse the outcome or the more morbidity was noted. Animals in infected control (Cohort 2) displayed significant distress with distinct signs of morbidity and infection (average score two weeks post-operative was 2.36 ± 0.85). All Cohort 2 animals had to be euthanized between 2–4 weeks per animal care guidelines. Animals in all remaining cohorts (Cohorts 1, 3–7) survived throughout the study as indicated by 100% survival (Fig. 2B). Importantly, animals in control uninfected (Cohort 1) implanted with a carved monolith of commercial HAP only displayed minimal symptoms of background surgical morbidity (average score two weeks post-operative was 0.17 ± 0.41). Animals implanted with antibiotic-eluting BVF polymer composite and inoculated with 10^7^ CFU *S*. *aureus* (compared to 10^5^ *S*. *aureus* used for the other cohorts) showed little to no morbidity (average score two weeks post-operative was 0.17 ± 0.38). Antibiotic controlled release (Cohort 7) was compared to intra-operatively HAP/BVF granules soaked in tobramycin (Cohort 5—average score two weeks post-operative was 1.08 ± 0.49) and placed in the defect site. Cohort 5 showed more morbidity than Cohort 7 polymer-controlled antibiotic release as indicated by the higher score (Fig. 2). Surprisingly, Cohort 4 (BVF with polymer but lacking antibiotic) showed signs of morbidity (average score two weeks post-operatively was 1.00 ± 0.00) but little to no mortality. Animal temperature and weight never varied beyond the standard deviation for all the study animals (data not shown), and only animals in Cohort 2 produced a positive mid-study culture (data not shown).

### Microbiology

**Fig 3:**
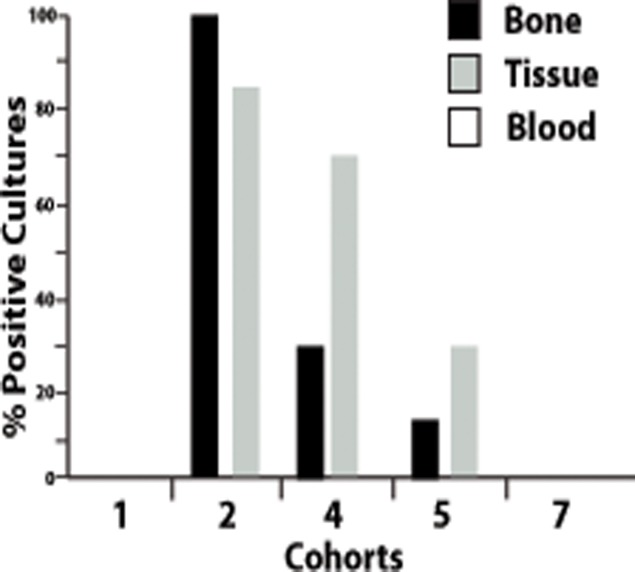
Microbiology assessments post euthanasia. Data represent the number of animals with culture positive swabs. No bacteria were detected in the blood of any animals. Only infected cohorts are shown with the exception of Cohort 1, which was included as a non-infection comparator. Note that Cohort 2 animals were euthanized prior to all other cohorts due to evident localized infection.

To verify that signs of infection and morbidity were attributed to a localized and not a systemic infection, blood was collected weekly and cultured for the presence of bacteria. All Cohort 2 animals were euthanized prior to the 8-week study endpoint (between 2 and 4 weeks) due to substantial localized infection. At necropsy, swabs of soft tissue and bone surrounding the surgical site were aseptically obtained and cultured (Fig. 3). Not surprisingly, every Cohort 2 animal showed significant bacterial counts from bone swabs, and the majority (85.7%) of animals were colony-positive from soft tissue swabs, although colonies were not counted. All colonies appeared to be homogenous *S*. *aureus* colonies. No colonies were detected in either the bone or soft tissue from Cohort 1 or Cohort 3 animals. Only a few animals in control Cohorts 4 and 5 were colony-positive from bone swabs (28.6% and 14.3% respectively) with additional positive cultures from soft tissue swabs (71.4% and 28.6% respectively). None of the animals included in antibiotic-eluting BVF Cohort 7 were colony-positive in either bone or the soft tissue, despite the substantially larger infectious *S*. *aureus* inoculum.

### Radiographic Evaluation

**Fig 4:**
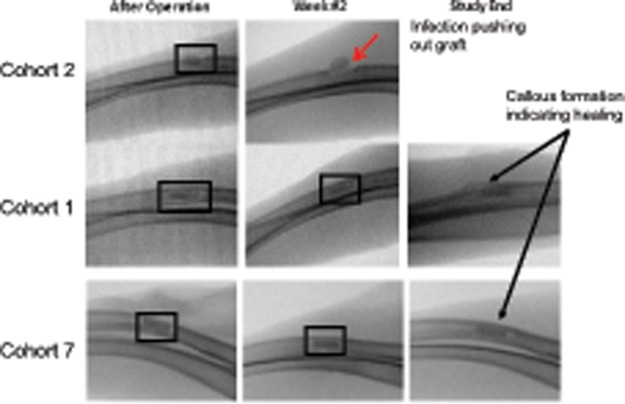
Radiological analysis and callus formation (Cohorts 1, 2, and 7). Only infectious Cohorts 2 and 7 are shown for comparison while Cohort 1 is included as a non-infection comparator. The presence of a distinct boney callus in Cohorts 1 and an incomplete bridging callus in Cohort 7 are indicative of bone healing. Although soft tissue inflammation cannot be quantitatively assessed, there appeared to be little or no soft tissue inflammation in Cohort 7 compared to Cohort 1, indicating a relatively normal bone healing response. Cohort 2 shows pronounced signs of infection as the device is displaced (indicated by a red arrow).

Bone response to the antibiotic-eluting BVF was assessed via weekly radiographic evaluations. All animals in Cohort 2 showed radiographic signs of infection and/or displacement of the implant as it was ejected from the surgical site into adjacent soft tissue (Fig. 4). Alternatively, animals in Cohort 1 showed no such radiographic evidence of device displacement, no evidence of significant soft tissue inflammation, and a slightly protruding boney callus formation. Animals in Cohort 7 also showed no device displacement, no overt signs of soft tissue inflammation, and only minimal signs of boney callus formation (Fig. 4). There was also radiolucency surrounding the Cohort 7 device that may be an additional indication of inadequate host bone ingrowth. Unfortunately, radiograph resolution was insufficient for complete analysis of bone-implant response.

### Histologic Evaluation

**Fig 5:**
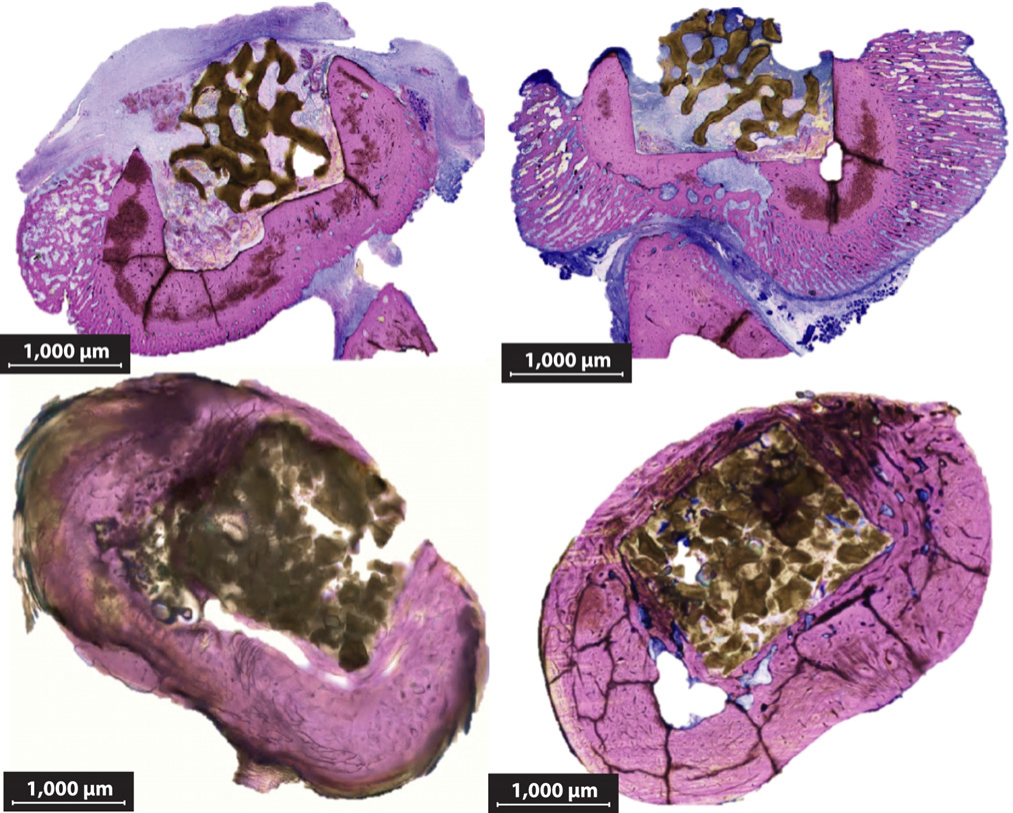
Sanderson’s Rapid Bone Stain (Cohorts 2 and 7). Two images are shown for each cohort to represent intrinsic variability in bone response to treatment. Soft tissue (blue), presumably fibrous capsule, immune cells, and infection are present in Cohort 2, but mostly lacking in Cohort 7. (2X magnification) BVF—Bone void filler, HB—Host bone. Insets are at 10X magnification.

**Fig 6:**
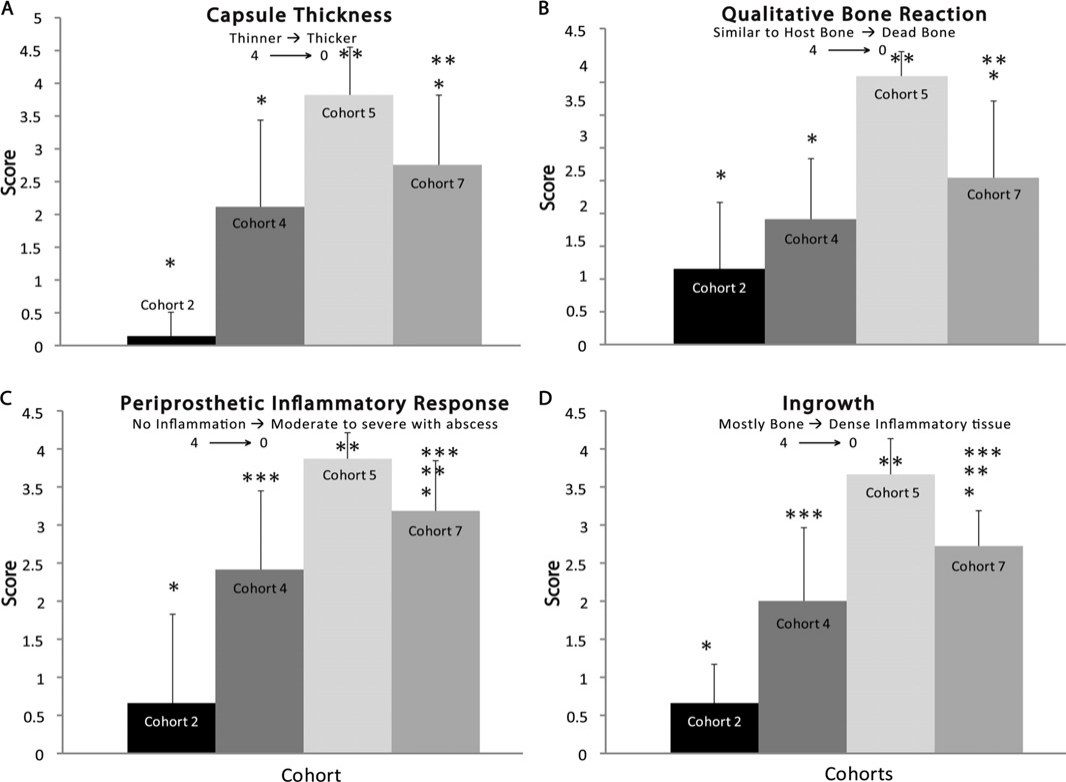
Semi-quantitative histology scoring. Histology images (20x) were obtained from each rabbit and assessed based on the capsule thickness (A), qualitative bone reaction (B), periprosthetic inflammation (C), and bone ingrowth (D). * significance p ≤ 0.05

Visual analysis of the dissected surgical site showed bridging callus formation in Cohort 1 and incomplete bridging in Cohorts 4 and 7, typical of normal bone healing (data not shown). Sanderson’s-stained histology sections (Fig. 5) corroborated the findings of the radiographic images, showing significant soft tissue inflammation and device displacement in Cohort 2 and more complete bone bridging with little soft tissue response in Cohort 7. Based on a published scoring system (Tables 2 and 3) [40, 42], Cohort 2 had 1) minimal capsule thickness (0.14 mm ± 0.36 mm), 2) significant osteoclastic resorption (1.07 ± 0.96), 3) minimal to mild inflammation with abscess (0.67 ± 1.18), and 4) dense inflammatory tissue surrounding the implant (0.47 ± 0.64) in the absence of the device polymer components (Fig. 6). Comparatively, Cohorts 4 and 7, which both had implants with a polymer matrix component, produced significantly different assessments when compared to Cohort 2, but not significantly different to each other when considering the quality of the bone (1.78 ± 0.88 and 2.38 ± 1.09 respectively, p = 0.052) or the capsule thickness (2.11 ± 1.32, 2.75 ± 1.06, respectively. p = 0.067), which was ≥100 μm and ≤150 μm. Cohort 4 did not produce significantly more inflammation compared to Cohort 7 (p = 0.02), but did produce less than Cohort 2 (p < 0.005) (2.44 ± 1.04 and 3.19 ± 0.66, respectively). Cohort 7 did have significantly different (p = 0.013) osseointegration (2.69 ± 0.048) compared to Cohort 4 (1.94 ± 1.06), but greater integration when compared to Cohort 2 (p < 0.005). Neither Cohort 4 nor 7 had substantial osseointegration evident at 8 weeks (Fig. 6). Cohort 3 showed no statistically significant differences (capsule thickness p = 0.45, qualitative bone response p = 0.92, periprosthetic inflammation p = 0.78, bone ingrowth p = 0.29) when compared to Cohort 4 on any metric measured and thus the majority of the Cohort 3 data are not presented for purposes of clarity and will be presented in a future manuscript. Cohort 6 animals were followed for 24 weeks to allow ample time for osseointegration; however, these results will be reported in a future manuscript, as this was the only cohort specifically designed to assess longer-term host bone integration. All data presented is deposited in a public repository that can be accessed at doi: 10.5061/dryad.q84m0.

## Discussion

The numerous BVFs currently used in bone repair remain susceptible to implant infection despite ongoing efforts to develop BVFs that locally release antibiotic [25, 35–37,44,45]. Local, extended antibiotic release to target pathogens at the BVF and prosthetic implant site minimizes development of antibiotic resistance by maintaining sustained drug amounts sufficient to eliminate microbial infection. Unfortunately, current designs that integrate local antibiotic release into BVF as a combination device suffer from large, early bolus drug release which not only risks local tissue toxicity in its magnitude, but exhibits bioactivity shorter than the clinically relevant 6–8 week window required to address senescent persistor organisms. Reliable infection control throughout the critical 6–8 week post-operative window routine for TJR hardware exchange as standard of care for two-stage revision procedures [25] is critical. These burst pharmacokinetics also leave patients susceptible to biofilm formation or development of antibiotic-resistant pathogens [10,25]. Thus, this work sought to develop an antibiotic-eluting BVF that 1) released antibiotic at therapeutic levels for 6–8 weeks, 2) was resorbable and osteoconductive, and 3) was able to integrate with host tissue. We hypothesized that local antibiotic release would target pathogens at the BVF site and would eliminate the periprosthetic bacterial burden, as well as provide an osteoconductive matrix for bone remodeling. For this to occur it was important that polymer resorption occur at a rate analogous to, or slightly faster than, the rate of host bone ingrowth. Based upon the observed and measured outcomes, this work corroborated previous *in vitro* work and demonstrated that the polymer-controlled, antibiotic-eluting BVF was able to eliminate a periprosthetic infection challenge of 10^7^ CFU *S*. *aureus in vivo*. Although animals implanted with the antibiotic-eluting bone void filler showed no signs of infection through 8 weeks, the device design failed to prove osteoconductive and integrated only minimally in 8 weeks with the surrounding host tissue as evidenced by histology.

Animals in control Cohort 1 showed no signs of infection, as expected, and validated the aseptic technique used for the surgical procedures. In contrast, all animals in Cohort 2 demonstrated signs of a positive and progressive infection and required euthanasia within the first 4 weeks following the procedure due to unresolved, localized, serious infection (composite score greater than 2.5—Fig. 2B). Additionally, Cohort 2 had the thickest fibrous capsule, most significant inflammation, lack of new bone growth, as well as significant bone abscess and dead bone (Fig. 6). Most significantly, when compared to Cohort 2, the rabbits that received the polymer-controlled, antibiotic-eluting BVF (Cohort 7) remained healthy throughout the 8-week study, as predicted based on the previous *in vitro* kinetic results [13,32], having significantly thinner fibrous capsule (p < 0.005), less inflammation (p < 0.005), and more bone ingrowth (p < 0.005) despite a significantly more adverse bone reaction (p < 0.005). In contrast, rabbits in the other deliberately infected cohorts (Cohorts 4 and 5) consistently exhibited clinical signs of infection, and/or showed culturable bacterial colonization of implants.

It was expected that Cohort 5 animals would show delayed onset infection, due to the lack of a polymer barrier and fast tobramycin diffusion away from the device. However, this was not observed (Fig. 2A). Based on the improved morbidity over time, it is thought that the initial burst of antibiotic was sufficient to support the rabbit’s substantial immune system [39,46] at an infectious burden of 10^5^ CFU. Such an effect is not anticipated with larger CFU inoculums. Animals in infected Cohort 4, lacking antibiotic, displayed initial signs of infection (average Petty score of 1, two weeks post-operatively, Fig. 2A) that eventually resolved. This may suggest some intrinsic antimicrobial properties for the BVF HAP/PCL/PEG matrix (Fig. 2). Based on previous *in vitro* studies that characterized the fabrication methodology [32], this device appeared to have a similar porosity and mechanical integrity as those implants that included antibiotic. An antimicrobial effect in this cohort was unexpected, and the polymer’s potential intrinsic antimicrobial properties are currently being explored.

All implants were placed immediately following pathogen inoculation in the marrow space. Intra-cohort evaluation of the consistency of the data suggested that time of incubation prior to device placement was not a significant contributor to inter-cohort differences. While *in vivo* kinetics and biodistribution of released drug were not determined, based on previous *in vitro* drug release kinetics [13,32] and literature reports [34] for this infection model, certain expectations were defined for each cohort; however, objectivity was retained via blinded scoring.

Although this study allowed for significant discoveries pertaining to the bactericidal nature of this antibiotic-eluting BVF, this study is not without its limitations. Most significantly, osseointegration was not achieved in the course of the 8-week study in Cohorts 4 or 7, as evidenced by a comparison with the bone response of the antibiotic-soaked BVF/HAP in Cohort 5, which displayed a thinner fibrous capsule, minimal inflammation, and more tissue ingrowth (Fig. 6). After eight weeks *in vivo*, Cohorts 4 and 7 revealed a thick fibrous capsule and minimal-to-no bone ingrowth (Figs. 5 and 6A). No significant difference in capsule thickness (p = 0.067) or qualitative bone response (p = 0.052) was detected between Cohorts 4 and 7 (Fig. 6).

Yeo et al. explored a PCL/tricalcium phosphate composite device implanted in the abdominal cavity of a rat over 24 weeks and noted the absence of foreign body giant cells, the hallmark of a chronic and pathological foreign body response, and a limited fibrous capsule. The apparent discrepancy between the limited fibrous encapsulation observed in this prior study [47] and the pronounced encapsulation observed in the current study could be a reflection of vast differences in the *in vivo* model, the PCL molecular weight, or the methodologies used for device fabrication. Nevertheless, an important feature of any implanted, degradable, polymer-controlled drug-releasing implant is the balance between implant degradation that enables appropriate drug pharmacokinetics to eliminate infection and host bone healing essential to the BVF [48,49]. Healing and bone regeneration must occur at rates analogous to device degradation to preserve bone structural integrity [50–52]. We posit that the slow PCL degradation *in vivo* [53,54] in this BVF form inhibits bone cell infiltration and subsequent host bone integration (Fig. 6D), and elicits the pronounced fibrous capsule formation in Cohorts 4 and 7.

Based on the preponderance of relatively inactive yellow marrow in the intermedullary canal of the older, larger animals used out of necessity in this study [55], it is possible that the 8-week time point represented an inadequate time frame in which to see normal bone turn-over and osseointegration. Hutmacher et al. considered bone mineralization and ingrowth into PCL scaffolds in both long-term (2 years) and short-term (15 weeks) *in vivo* model systems as an indication of biocompatibility, finding limited bone ingrowth within 15 weeks [56,57]. Additionally, Miyai et al. observed limited new bone formation at the surface of their antibiotic releasing PCL/β-tricalcium phosphate device after 4 weeks in a rabbit mandible osteomyelitis model with significant ingrowth after 50 weeks [58]; however, they observed limited infection control. Both of these studies produced similar results at their early time points when compared to the new bone growth observed in this study. Nevertheless, polymeric/HAP composite components in future device formulations can readily be altered to accelerate the matrix resorption, facilitating improved host bone integration. Furthermore, the observed high burst rate and fractional load of antibiotic is known to hinder the ability of osteoblasts to initiate bone remodeling [59]. Preliminary *in vivo* cytotoxicity experiments (not shown) do not support significant toxicity to cultured osteoblasts from this BVF drug release. Therefore, the actual *in vivo* host tissue response to these release kinetics and to this antibiotic are critical to the bone regenerative potential of this composite BVF device beyond its antimicrobial efficacy.

## Conclusion

While all of the BVF device components described (i.e., HAP/calcium carbonate granules, PCL and PCL polymer blends, antibiotic) have an extensive history of preclinical and clinical use [60], comparisons with other *in vivo* models exploring their use is of limited comparative value in the context of this new antibiotic BVF device. The BVF combination device described here represents a second-generation antibiotic-releasing bone void filler [13–15,32,60] with improved molded fabrication (i.e., consistency, ease, etc.) and enhanced drug delivery and antimicrobial activity (e.g., for tobramycin, a release duration greater than 10-weeks *in vitro*). In agreement with previous *in vitro* studies demonstrating extended bactericidal activity [13,32], *in vivo* implantation of the antibiotic-eluting BVF was able to eradicate the infection challenge throughout the 8-week post surgery experimental time course, meeting our first objective even in the presence of a rigorous, persistent local *S*. *aureus* infectious challenge. Furthermore, in spite of inadequate osseointegration, the BVF platform described remains versatile enough to adapt to the constraints of the *in vivo* environment and can accommodate different antibiotics and drugs for release, even in combinations [32,61,62]. Future work will focus on improved osseointegration while maintaining the demonstrated anti-infective efficacy.

## References

[1] Lee K, Goodman SB (2008) Current state and future of joint replacements in the hip and knee. Expert Rev Med Devices 5: 383–393. 10.1586/17434440.5.3.383 18452388

[2] Research and Markets: The Future of Orthopeadic Implants, Analysis and Forecasts to 2016—Joint Reconstruction and Spinal Implants Creating Growth Opportunities | Business Wire (2010). Available: http://www.businesswire.com/news/home/20100615006333/en/Research-Markets-Future-Orthopeadic-Implants-Analysis-Forecasts#.U3pVb8ZjBuY. Accessed 19 May 2014.

[3] Gunnam R (2010) The future of orthopedic implants analysis and forecasts to 2016. GBI Research.

[4] Kurtz SM, Lau E, Ong K, Zhao K, Kelly M, Bozic KJ (2009) Future young patient demand for primary and revision joint replacement: national projections from 2010 to 2030. Clin Orthop 467: 2606–2612. 10.1007/s11999-009-0834-6 19360453 PMC2745453

[5] Bostrom MP, Seigerman DA (2005) The clinical use of allografts, demineralized bone matrices, synthetic bone graft substitutes and osteoinductive growth factors: a survey study. HSS J 1: 9–18. 10.1007/s11420-005-0111-5 18751803 PMC2504134

[6] Surgeons (2013) Joint Revision Surgery—When Do I Need It? Available: http://orthoinfo.aaos.org/topic.cfm?topic=A00510. Accessed 21 May 2013.

[7] Kurtz SM, Lau E, Schmier J, Ong KL, Zhao K, Parvizi J (2008) Infection burden for hip and knee arthroplasty in the United States. J Arthroplasty 23: 984–991. 10.1016/j.arth.2007.10.017 18534466

[8] Conterno LO, da Silva Filho CR (2009) Antibiotics for treating chronic osteomyelitis in adults. Cochrane Database Syst Rev 3. Available: http://onlinelibrary.wiley.com/doi/10.1002/14651858.CD004439.pub2/pdf/standard. Accessed 26 June 2013. 10.1002/14651858.CD004439.pub2 19588358

[9] Landersdorfer CB, Bulitta JB, Kinzig M, Holzgrabe U, Sörgel F (2009) Penetration of Antibacterials into Bone. Clin Pharmacokinet 48: 89–124. 10.2165/0003088-200948020-00002 19271782

[10] Campoccia D, Montanaro L, Speziale P, Arciola CR (2010) Antibiotic-loaded biomaterials and the risks for the spread of antibiotic resistance following their prophylactic and therapeutic clinical use. Biomaterials 31: 6363–6377. 10.1016/j.biomaterials.2010.05.005 20542556

[11] Hetrick EM, Schoenfisch MH (2006) Reducing implant-related infections: active release strategies. Chem Soc Rev 35: 780–789. 10.1039/b515219b 16936926

[12] Trampuz A, Widmer AF (2006) Infections associated with orthopedic implants. Curr Opin Infect Dis 19: 349–356. 10.1097/01.qco.0000235161.85925.e8 16804382

[13] Brooks AE, Brooks BD, Davidoff SN, Hogrebe PC, Fisher MA, Grainger DW (2013) Polymer-controlled release of tobramycin from bone graft void filler. Drug Deliv Transl Res 3: 518–530. 10.1007/s13346-013-0155-x 25786372

[14] Davidoff SN, Call BP, Hogrebe PC, Grainger DW, Brooks AE (2010) A robust method to coat allograft bone with a drug-releasing polymer shell-biomed 2010. Biomed Sci Instrum 46: 184 20467092

[15] Sevy JO, Slawson MH, Grainger DW, Brooks AE (2010) Assay method for polymer-controlled antibiotic release from allograft bone to target orthopaedic infections-biomed 2010. Biomed Sci Instrum 46: 136 20467084

[16] Kluin OS, van der Mei HC, Busscher HJ, Neut D (2013) Biodegradable vs non-biodegradable antibiotic delivery devices in the treatment of osteomyelitis. Expert Opin Drug Deliv 10: 341–351. 10.1517/17425247.2013.751371 23289645

[17] Neut D, Van De Belt H, Stokroos I, Van Horn JR, Van Der Mei HC, Busscher HJ (2001) Biomaterial-associated infection of gentamicin-loaded PMMA beads in orthopaedic revision surgery. J Antimicrob Chemother 47: 885–891. 11389124 10.1093/jac/47.6.885

[18] Neut D, van de Belt H, van Horn JR, van der Mei HC, Busscher HJ (2003) The effect of mixing on gentamicin release from polymethylmethacrylate bone cements. Acta Orthop Scand 74: 670–676. 10.1080/00016470310018180 14763697

[19] Neut D, van de Belt H, van Horn JR, van der Mei HC, Busscher HJ (2003) Residual gentamicin-release from antibiotic-loaded polymethylmethacrylate beads after 5 years of implantation. Biomaterials 24: 1829–1831. 12593965 10.1016/s0142-9612(02)00614-2

[20] Van de Belt H, Neut D, Schenk W, van Horn JR, van der Mei HC, Busscher HJ (2000) Gentamicin release from polymethylmethacrylate bone cements and Staphylococcus aureus biofilm formation. Acta Orthop Scand 71: 625–629. 10.1080/000164700317362280 11145392

[21] Anagnostakos K, Fürst O, Kelm J (2006) Antibiotic-impregnated PMMA hip spacers: current status. Acta Orthop 77: 628–637. 16929441 10.1080/17453670610012719

[22] Tan HL, Lin WT, Tang TT (2012) The use of antimicrobial-impregnated PMMA to manage periprosthetic infections: controversial issues and the latest developments. Int J Artif Organs 35: 832–839. 10.5301/ijao.5000163 23138709

[23] Arciola CR, Campoccia D, Montanaro L (2002) Effects on antibiotic resistance of Staphylococcus epidermidis following adhesion to polymethylmethacrylate and to silicone surfaces. Biomaterials 23: 1495–1502. 11829446 10.1016/s0142-9612(01)00275-7

[24] DiMaio FR, O’Halloran JJ, Quale JM (1994) In vitro elution of ciprofloxacin from polymethylmethacrylate cement beads. J Orthop Res Off Publ Orthop Res Soc 12: 79–82. 10.1002/jor.1100120110 8113945

[25] Brooks BD, Brooks AE, Grainger DW (2013) Antimicrobial Medical Devices in Preclinical Development and Clinical Use In: Moriarty TF, Zaat SAJ, Busscher HJ, editors. Biomaterials Associated Infection. Springer New York pp. 307–354. Available: http://www.springerlink.com/content/rp22657g55495648/abstract/. Accessed 30 October 2012.

[26] Giannoudis PV, Dinopoulos H, Tsiridis E (2005) Bone substitutes: an update. Injury 36: S20–S27. 16188545 10.1016/j.injury.2005.07.029

[27] Valle AGD, Bostrom M, Brause B, Harney C, Salvati EA (2001) Effective bactericidal activity of tobramycin and vancomycin eluted from acrylic bone cement. Acta Orthop 72: 237–240. 11480597 10.1080/00016470152846547

[28] Ketonis C, Barr S, Adams CS, Hickok NJ, Parvizi J (2010) Bacterial colonization of bone allografts: establishment and effects of antibiotics. Clin Orthop 468: 2113–2121. 10.1007/s11999-010-1322-8 20361282 PMC2895848

[29] Richards RG, Moriarty TF, Miclau T, McClellan RT, Grainger DW (2012) Advances in Biomaterials and Surface Technologies. J Orthop Trauma 26: 703–707. 10.1097/BOT.0b013e31826e37a2 22913967

[30] Vasilev K, Cook J, Griesser HJ (2009) Antibacterial surfaces for biomedical devices. Expert Rev Med Devices 6: 553–567. 10.1586/erd.09.36 19751126

[31] Simchi A, Tamjid E, Pishbin F, Boccaccini AR (2011) Recent progress in inorganic and composite coatings with bactericidal capability for orthopaedic applications. Nanomedicine Nanotechnol Biol Med 7: 22–39. 10.1016/j.nano.2010.10.005 21050895

[32] Brooks B.D., Sinclair K., Davidoof S.N., Williams A., Lawson S, Williams AG, et al (2014) Molded Polymer-Coated Composite Bone Void Filler Improves Tobramycin Controlled Release Kinetics. J Biomed Mater Res Part B—Appl Biomater 102:1074–1083. 24376164 10.1002/jbm.b.33089

[33] Kanellakopoulou K, Giamarellos-Bourboulis EJ (2000) Carrier systems for the local delivery of antibiotics in bone infections. Drugs 59: 1223–1232. 10882159 10.2165/00003495-200059060-00003

[34] Koort JK, Suokas E, Veiranto M, Mäkinen TJ, Jalava J, Tormala P, et al (2006) In vitro and in vivo testing of bioabsorbable antibiotic containing bone filler for osteomyelitis treatment. J Biomed Mater Res A 78: 532–540. 16736479 10.1002/jbm.a.30766

[35] McKee MD, Wild LM, Schemitsch EH, Waddell JP (2002) The use of an antibiotic-impregnated, osteoconductive, bioabsorbable bone substitute in the treatment of infected long bone defects: early results of a prospective trial. J Orthop Trauma 16: 622–627. 12368641 10.1097/00005131-200210000-00002

[36] Nandi SK, Roy S, Mukherjee P, Kundu B, De DK, Basu D (2010) Orthopaedic applications of bone graft & graft substitutes: a review. Available: http://imsear.hellis.org/handle/123456789/135534. Accessed 26 June 2013. 20693585

[37] Winkler H, Stoiber A, Kaudela K, Winter F, Menschik F (2008) One stage uncemented revision of infected total hip replacement using cancellous allograft bone impregnated with antibiotics. J Bone Joint Surg Br 90: 1580–1584. 10.1302/0301-620X.90B12.20742 19043128

[38] Koort JK, Mäkinen TJ, Suokas E, Veiranto M, Jalava J, Knuuti J, et al (2005) Efficacy of Ciprofloxacin-Releasing Bioabsorbable Osteoconductive Bone Defect Filler for Treatment of Experimental Osteomyelitis Due to Staphylococcus aureus. Antimicrob Agents Chemother 49: 1502–1508. 10.1128/AAC.49.4.1502-1508.2005 15793132 PMC1068592

[39] Smeltzer MS, Thomas JR, Hickraon SG, Skinner RA, Nelson CL, Griffith D, et al (1997) Characterization of a rabbit model of staphylococcal osteomyelitis. J Orthop Res 15: 414–421. 10.1002/jor.1100150314 9246088

[40] Sinclair KD, Pham TX, Williams DL, Farnsworth RW, Loc-Carrillo CM, Bloebaum RD (2013) Model development for determining the efficacy of a combination coating for the prevention of perioperative device related infections: a pilot study. J Biomed Mater Res B Appl Biomater 101: 1143–1153. 10.1002/jbm.b.32924 23564717

[41] Isaacson BM, Brown AA, Brunker LB, Higgins TF, Bloebaum RD (2011) Clarifying the Structure and Bone Mineral Content of Heterotopic Ossification. J Surg Res 167: e163–e170. 10.1016/j.jss.2010.12.047 21392799

[42] Checketts RG, MacEachem AG, Otterbum M (2000) Pin Track Infection and the Principles of Pin Site Care In: Bastiani GD, Apley AG, FFPM AGM MPharm, editors. Orthofix External Fixation in Trauma and Orthopaedics. Springer London pp. 97–103. Available: http://link.springer.com/chapter/10.1007/978-1-4471-0691-3_11. Accessed 29 January 2014.

[43] An YH, Kang QK, Arciola CR (2006) Animal models of osteomyelitis. Int J Artif Organs 29: 407–420. 16705610 10.1177/039139880602900411

[44] Ketonis C, Barr S, Shapiro IM, Parvizi J, Adams CS, Hickok NJ (2011) Antibacterial activity of bone allografts: comparison of a new vancomycin-tethered allograft with allograft loaded with adsorbed vancomycin. Bone 48: 631–638. 10.1016/j.bone.2010.10.171 21035576 PMC3039041

[45] Winkler H, Janata O, Berger C, Wein W, Georgopoulos A (2000) In vitro release of vancomycin and tobramycin from impregnated human and bovine bone grafts. J Antimicrob Chemother 46: 423–428. 10980169 10.1093/jac/46.3.423

[46] Worlock P, Slack R, Harvey L, Mawhinney R (1988) An experimental model of post-traumatic osteomyelitis in rabbits. Br J Exp Pathol 69: 235–244. 3377964 PMC2013214

[47] Yeo A, Rai B, Sju E, Cheong J j., Teoh S h. (2008) The degradation profile of novel, bioresorbable PCL—TCP scaffolds: An in vitro and in vivo study. J Biomed Mater Res A 84A: 208–218. 10.1002/jbm.a.31454 17607768

[48] Mouriño V, Cattalini JP, Roether JA, Dubey P, Roy I, Boccaccini AR (2013) Composite polymer-bioceramic scaffolds with drug delivery capability for bone tissue engineering. Expert Opin Drug Deliv 10: 1353–1365. 10.1517/17425247.2013.808183 23777443

[49] Rezwan K, Chen QZ, Blaker JJ, Boccaccini AR (2006) Biodegradable and bioactive porous polymer/inorganic composite scaffolds for bone tissue engineering. Biomaterials 27: 3413–3431. 10.1016/j.biomaterials.2006.01.039 16504284

[50] Coombes AGA, Meikle MC (1994) Resorbable synthetic polymers s replacements for bone graft. Clin Mater 17: 35–67. 10.1016/0267-6605(94)90046-9 10150176

[51] Hutmacher DW (2000) Scaffolds in tissue engineering bone and cartilage. Biomaterials 21: 2529–2543. 10.1016/S0142-9612(00)00121-6 11071603

[52] Holmbom J, Södergård A, Ekholm E, Märtson M, Kuusilehto A, Saukko P, et al (2005) Long-term evaluation of porous poly(ϵ-caprolactone-co-L-lactide) as a bone-filling material. J Biomed Mater Res A 75A: 308–315. 10.1002/jbm.a.30418 16059893

[53] Woodruff MA, Hutmacher DW (2010) The return of a forgotten polymer—Polycaprolactone in the 21st century. Prog Polym Sci 35: 1217–1256. 10.1016/j.progpolymsci.2010.04.002

[54] Ekholm M, Hietanen J, Lindqvist C, Rautavuori J, Santavirta S, Suuronen R (1999) Histological study of tissue reactions to ε-caprolactone—lactide copolymer in paste form. Biomaterials 20: 1257–1262. 10.1016/S0142-9612(97)00080-X 10403042

[55] Hernigou P, Poignard A, Manicom O, Mathieu G, Rouard H (2005) The use of percutaneous autologous bone marrow transplantation in nonunion and avascular necrosis of bone. J Bone Joint Surg Br 87-B: 896–902. 10.1302/0301-620X.87B7.16289 15972899

[56] Sawyer AA, Song SJ, Susanto E, Chuan P, Lam CXF, Woodruff MA, et al (2009) The stimulation of healing within a rat calvarial defect by mPCL-TCP/collagen scaffolds loaded with rhBMP-2. Biomaterials 30: 2479–2488. 10.1016/j.biomaterials.2008.12.055 19162318

[57] Evans RP, Nelson CL, Harrison BH, Sherk HH (2005) THE CLASSIC: The Effect of Wound Environment on the Incidence of Acute Osteomyelitis. Clin Orthop 439: 4–9. 16205127 10.1097/01.blo.0000183276.33237.24

[58] Miyai T, Ito A, Tamazawa G, Matsuno T, Sogo Y, Nakamura C, et al (2008) Antibiotic-loaded poly-ε-caprolactone and porous β-tricalcium phosphate composite for treating osteomyelitis. Biomaterials 29: 350–358. 10.1016/j.biomaterials.2007.09.040 17977596

[59] Antoci V, Adams CS, Hickok NJ, Shapiro IM, Parvizi J (2007) Antibiotics for Local Delivery Systems Cause Skeletal Cell Toxicity In Vitro: Clin Orthop 462: 200–206. 10.1097/BLO.0b013e31811ff866 17572634

[60] Porter JR, Ruckh TT, Popat KC (2009) Bone tissue engineering: A review in bone biomimetics and drug delivery strategies. Biotechnol Prog: NA—NA. 10.1002/btpr.246 19824042

[61] Brooks BD, Davidoff SN, Grainger DW, Brooks AE (2013) Comparisons of release of several antibiotics from antimicrobial polymer-coated allograft bone void filler. Int J Biomed Mater Res 1: 21–25.

[62] DeGooyer Jason, Davidoff Sherry N., Brooks Benjamin D., Grainger David W., Brooks Amanda E. (2014) Polymer-controlled extended combination release of silver and chlorhexidine from a bone void filler. Biomed Sci Instrum 50: 47–53. 25405403

